# Genome editing of human pancreatic beta cell models: problems, possibilities and outlook

**DOI:** 10.1007/s00125-019-4908-z

**Published:** 2019-06-03

**Authors:** Diego Balboa, Rashmi B. Prasad, Leif Groop, Timo Otonkoski

**Affiliations:** 1 Stem Cells and Metabolism Research Program, Faculty of Medicine, University of Helsinki, PO Box 63, (Haartmaninkatu 8), FI-00014 Helsinki, Finland; 2 Present Address: Centre for Genomic Regulation (CRG), The Barcelona Institute of Science and Technology, Barcelona, Spain; 3 Genomics, Diabetes and Endocrinology, Lund University Diabetes Centre, CRC, Malmö, Sweden; 4 Institute of Molecular Medicine, Centre of Excellence in Complex Disease Genetics, University of Helsinki, Helsinki, Finland; 5 Children’s Hospital, Helsinki University Hospital and University of Helsinki, Helsinki, Finland

**Keywords:** Beta cells, Cell models, CRISPR-Cas9, Diabetes, Genome editing, Human islets, Pancreas, Review, Stem cells

## Abstract

Understanding the molecular mechanisms behind beta cell dysfunction is essential for the development of effective and specific approaches for diabetes care and prevention. Physiological human beta cell models are needed for this work. We review the possibilities and limitations of currently available human beta cell models and how they can be dramatically enhanced using genome-editing technologies. In addition to the gold standard, primary isolated islets, other models now include immortalised human beta cell lines and pluripotent stem cell-derived islet-like cells. The scarcity of human primary islet samples limits their use, but valuable gene expression and functional data from large collections of human islets have been made available to the scientific community. The possibilities for studying beta cell physiology using immortalised human beta cell lines and stem cell-derived islets are rapidly evolving. However, the functional immaturity of these cells is still a significant limitation. CRISPR-Cas9 (Clustered Regularly Interspaced Short Palindromic Repeats/CRISPR-associated protein 9) has enabled precise engineering of specific genetic variants, targeted transcriptional modulation and genome-wide genetic screening. These approaches can now be exploited to gain understanding of the mechanisms behind coding and non-coding diabetes-associated genetic variants, allowing more precise evaluation of their contribution to diabetes pathogenesis. Despite all the progress, genome editing in primary pancreatic islets remains difficult to achieve, an important limitation requiring further technological development.

**Electronic supplementary material:**

The online version of this article (10.1007/s00125-019-4908-z) contains a slide of the figure for download, which is available to authorised users.

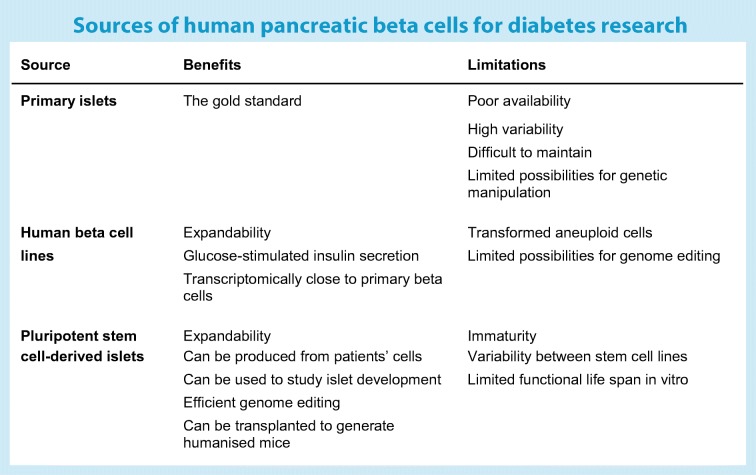



## Introduction

Failing beta cell function is a major culprit in all forms of diabetes. In type 1 diabetes this results from an interaction between genetic predisposition and environmental factors, culminating in an immune-mediated loss of beta cells. In monogenic diabetes, insulin secretion is deficient entirely based on mutations in genes that are important for beta cell function. Type 2 diabetes is considered to result from a collision between genetic predisposition and an affluent environment, which means that type 2 diabetes develops when people no longer can increase their insulin secretion to meet the increased demands imposed by obesity and insulin resistance. Not surprisingly, most of the 403 genetic variants identified in genome-wide association studies (GWAS) to be associated with type 2 diabetes [1] have been shown to influence beta cell function. A problem with these studies is that they have considered type 2 diabetes as a relatively homogenous disease. We have recently shown that this is not the case. By measuring a few pathogenically relevant variables, we could break down type 2 diabetes into five subgroups with quite different characteristics and disease progression [2].

Variants in several genes show strong association with type 2 diabetes risk, including those in *TCF7L2*, *SLC30A8* and *MTNR1B* [1]. Although the genetic risk of type 1 diabetes is most strongly associated with the HLA genes, more than 50 additional genes or loci have been associated with the disease, most being expressed in the pancreatic beta cells [3]. However, it is not easy to infer causality from a common genetic variant associated with either type 1 or type 2 diabetes. Therefore, functional studies using genetically defined cells in appropriate models are required. Possibilities for studying human beta cell function in vivo are limited. In order to understand the pathogenic role of diabetes-associated genetic variants, experimental beta cell models are needed. Rodent models, particularly transgenic mice, have provided a lot of valuable information but they have limitations due to obvious genetic and physiological species differences. Essentially, there are three possible ways to study human beta cells directly: (1) primary islets isolated from the pancreas of organ donors; (2) clonal human beta cell lines and (3) islet-like cells differentiated from human pluripotent stem cells (hPSCs), comprising either human embryonic stem cells (hESCs) or human induced pluripotent stem cells (hiPSCs) (see Text box).

## Primary human islets

Human pancreatic islets obtained from organ donor pancreases or from pancreatic surgery are very informative, since they are obtained while the blood flow is still intact, thereby retaining functionality of the cells. Comprehensive transcriptomic profiling of such islets, together with GWAS, has facilitated extensive analysis of expression [4] and effects of genetic variation on gene expression (i.e. expression quantitative traits [eQTLs], splicing [splice QTLS], allelic imbalance [5], *cis*-regulatory networks [6, 7] and non-coding RNAs [8]) using collections of isolated human islets. This has enabled the discovery of numerous genes with a potential role in glucose metabolism and insulin secretion. In order to make these resources more accessible to the scientific community, the Islet Gene View was created, providing comprehensive information on gene expression in relation to diabetes status, insulin secretion, expression of other pancreatic genes and related phenotypes of interest [9]. Overlaying expression data with data on regions of open chromatin (DNase I hypersensitive sites sequencing [DNase-seq], assay for transposase-accessible chromatin sequencing **[**ATAC-seq]) [10], histone modifications (chromatin immunoprecipitation sequencing [ChIP-seq]) [11] and spatial chromatin organisation data (Hi-C, Capture-C or 4-C methods) [12] can facilitate a better understanding of the genomic regulation critical for appropriate islet function.

Pancreatic islets consist of multiple cell types, each with distinctive functions. Performing single-cell mRNA sequencing on different cell types, including alpha, beta, gamma, delta and epsilon cells from adult and fetal pancreases, can facilitate the identification of unique cell-specific expression profiles [13–15] in the hope of distinguishing profiles between type 2 diabetes and non-diabetic donors [16, 17]. Interestingly, key type 2 diabetes genes reported from previous studies, such as *TCF7L2* and others, were missing in these data, suggesting that these studies may have been underpowered or that some of the earlier studies using bulk RNA sequencing may have been confounded by signals from cells other than endocrine cells. In addition, these differences are likely to reflect the technical limitations of single-cell mRNA sequencing technologies: limited number of cells analysed and a low gene detection rate.

Different viral vectors have been exploited to perform overexpression and perturbation experiments in human islets. Lentiviruses, adenovirus and adeno-associated viruses (AAVs) carrying cDNA-expressing constructs or short hairpin RNA (shRNA) have been transduced to human islet cells [7]. However, genome editing using site-directed endonucleases in primary islets has not previously been reported, possibly because this approach may be challenging due to a variety of factors, including poor delivery efficiency to intact islets, the quiescent nature of the cells or the sensitivity of the cells to these manipulations. These limitations might be overcome in the future with use of optimised Clustered Regularly Interspaced Short Palindromic Repeats (CRISPR)CRISPR-associated protein 9 (Cas9) approaches, such as those tailored for primary cells (e.g. Guide Swap [18]), the use of Cas9 base editors [19] or improved delivery methods to intact islets (e.g. smaller Cas9 delivered using AAVs). An alternative possibility would be the use of bioengineered human pseudoislets [20], in which dissociated cells are treated with CRISPR-Cas9 and then reaggregated.

## Human beta cell lines

Human beta cell lines have been a long-sought resource for diabetes research. Finally, Scharfmann and co-workers succeeded in generating stable human beta cell lines from human fetal pancreatic cells using the SV40LT oncogene under the insulin promoter [21]. The first line, EndoC-βH1, has now been adopted for use in many laboratories and generally accepted as a stable glucose-responsive human beta cell line, which has numerous applications, ranging from studies of insulin secretion to studies of beta cell damage [22]. The line has obvious advantages, such as the possibility to expand it in an unlimited manner and its responsiveness to glucose at a physiological range. Additional EndoC-βH lines 2 and 3 have been developed in which the oncogene can be removed, resulting in cell-cycle arrest and increased insulin secretion in response to glucose. EndoC-βH cells are amenable to different perturbation experiments since they can be transfected chemically and electroporated with plasmid vectors or small interfering RNA (siRNA) molecules, or transduced with viral vectors [22]. However, it is challenging to genetically modify this cell line at a clonal level, given its slow growth rate and low clonal efficiency. Furthermore, it should be remembered that these are transformed aneuploid cells that cannot be taken as a direct counterpart of the primary beta cell.

## hPSC-based models

The third option for achieving human beta cells for experimental studies is based on the differentiation of hPSCs. The first report describing successful differentiation of hESCs to pancreatic endocrine cells was published in 2006 by D’Amour et al [23]. Since then, the stepwise protocols that are needed to mimic normal pancreatic differentiation have been further optimised, resulting in methods that lead to the generation of large numbers of islet-like cell aggregates consisting predominantly of beta cells that are capable of responding to physiological insulin secretagogues [24, 25]. Metabolic maturation of the cells, measured as robust glucose-stimulated insulin secretion, is still difficult to achieve in vitro, but the immature cells do have a remarkable capacity for maturation after implantation into rodents. This enables the generation of ‘humanised’ mouse models, where the implanted human beta cells are responsible for glycaemic control in the mouse. We have recently reviewed the possibilities of stem cell strategies for the modelling of beta cell pathophysiology elsewhere [26]. Organoid technologies have evolved rapidly, enabling the generation of self-renewing ‘mini-organs’ from both primary tissue stem cells and pluripotent stem cells [27]. Pancreatic organoids have also been described, although this technique remains unproven as a practical solution for the efficient expansion and differentiation of pancreatic progenitors.

**Fig. 1:**
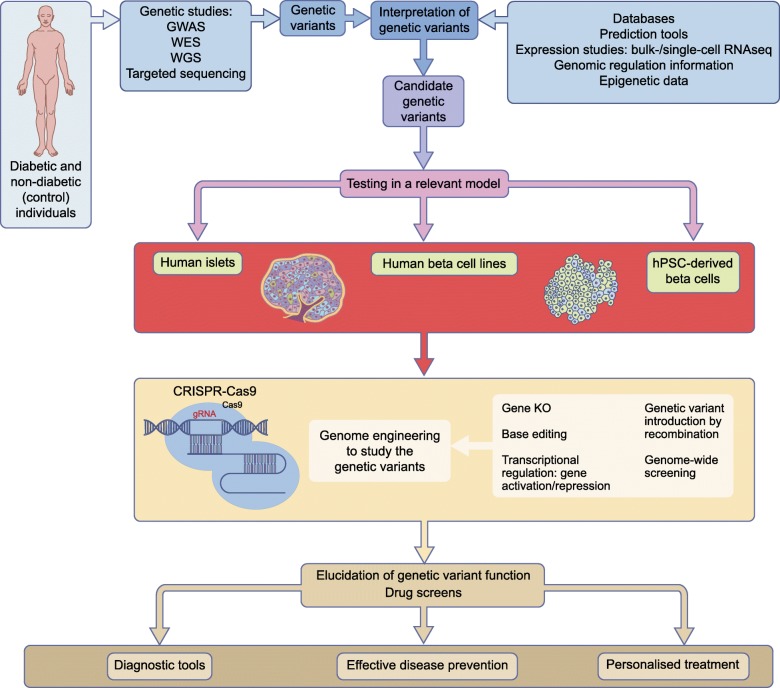
The central role of human beta cell models in the functional analysis of diabetes-associated genotypes. Genetic variants identified in people affected with diabetes (via genetic studies) can be interpreted using integrated functional genomic data (databases, prediction tools, epigenetic data, etc.). Candidate genetic variants require validation in relevant experimental models. Genome engineering technologies (e.g. gene knockout, base editing and genetic recombination) facilitate the genetic manipulation of cellular models to elucidate the role of the candidate genetic variants. In particular, genome engineering using CRISPR-Cas9 systems (consisting of two parts: a Cas9 endonuclease protein and gRNAs) have recently opened exciting new avenues for interrogating the functional impact of diabetes-associated genetic variants. These genome engineered models might also be utilised as scalable drug-screening platforms. Understanding the functional impact of diabetes-associated genetic variants will allow better diagnosis and stratification of diabetes cases, implementation of more effective interventions for diabetes prevention and more optimal personalised treatment for people affected by diabetes. KO, knockout; RNAseq, RNA sequencing; WES, whole exome sequencing; WGS, whole genome sequencing. This figure is available as a downloadable slide

The use of stem cells to generate human beta cells is an optimal approach for several reasons (see Text box); a key advantage is the possibility of using patient-derived iPSCs as starting material, making it possible to recapitulate functional features that are specific for an individual’s genotype. The recapitulation of normal organ development also allows us to model developmental defects, which is not possible if end-stage differentiated islets of beta cells are used. However, because of the high variability between individual iPSC lines, findings need to be replicated in a large number of lines derived from different donors and an equally large number of control individuals [28]. Considering the demanding differentiation procedures, this is a formidable challenge. Therefore, genome editing of stem cells is an attractive possibility for reducing the variability between cell lines and focusing on the impact of specific genetic variants (Fig. 1). Different genome engineering technologies, like zinc finger nucleases (ZFN) [29] and transcription activator like effector nucleases (TALEN) [30], have been used successfully on hPSCs but these have been superseded by CRISPR-Cas9 technology, which has dramatically improved the possibilities of genome editing.

## Genome editing with CRISPR-Cas9

Genome engineering is based on the use of sequence-specific endonucleases that introduce DNA double-strand breaks in a targeted genomic sequence. These targeted cuts may disrupt that particular sequence, possibly resulting in a gene knockout, or stimulate homologous recombination with exogenous DNA templates (homology-directed repair [HDR]), which can be exploited to create knockins in order to correct or introduce point mutations.

The genome editing revolution started in 2013, when the first CRISPR-Cas9 system was engineered to work in mammalian cells [31–33]. The system consists of two parts: a Cas9 endonuclease protein and short RNA molecules, called guide RNAs (gRNAs). The latter are loaded into the Cas9 protein to form a ribonucleoprotein complex that seeks and cleaves DNA sequences complementary to the gRNA sequence. CRISPR-Cas9 systems have been widely used to disrupt genes in different cell lines and organisms. CRISPR technology and its multiple applications have been thoroughly reviewed elsewhere [34, 35].

The overall gene disruption efficiency depends on many factors, including the expression level of Cas9 protein, the sequence and quality features of the gRNA and the cell-cycle phase. To generate a reliable gene knockout, gRNAs should be designed to target all the splice variants of the gene of interest, including essential functional domains and considering possible alternative translation start sites. Targeting regions directly downstream of the start codon is also used but this may result in hypomorphic alleles due to alternative start sites. Defined genomic regions can also be excised by using pairs of gRNAs to generate a large deletion. This is particularly useful for studying the importance of non-coding and regulatory elements [12, 36]. The generation of large deletions in the genome might have unintended consequences if, for example, non-annotated regulatory regions are also removed. For this reason, CRISPR-based genome manipulation experiments should be carefully planned to include appropriate controls and alternative modification approaches (e.g. point mutation introduction, base editing).

The introduction or correction of particular point mutations using CRISPR-Cas9 is an important application by which to investigate the role of particular genetic variants. To achieve this, a Cas9-mediated cut is generated adjacent to the position of interest while providing a homologous donor template with the intended nucleotide change, usually in the form of a short single-stranded DNA oligo, that will recombine by HDR [37]. Another approach is the use of engineered Cas9 versions that work as base editors, which convert DNA bases (transitions C to T and A to G) without cleaving the DNA, thereby avoiding the risks of undesired on- and off-target cut effects [19]. An interesting advantage of base editing is its high efficiency in quiescent cells [38], in contrast to the inefficiency of HDR in non-dividing cells. This could be exploited to manipulate adult human beta cells, which are largely quiescent.

Exciting novel experimental possibilities have been enabled by the engineering of catalytically inactive (‘dead’) Cas9 proteins with transcriptional activator and repressor domains (e.g. repeats of VP16, Krüppel associated box [KRAB], or epigenetic modulators such as DNA methyltransferase 3α [DNMT3A], p300, etc.) [39, 40]. These Cas9-based effectors make it possible to perform hitherto unfeasible targeted transcriptional modulation and epigenome modification of endogenous loci. One conceptual advance has been the development of unbiased CRISPR-Cas9-based whole-genome genetic screenings, enabling genome-wide-scale gene knockouts, deletion of regulatory regions and transcriptional activation or repression [41].

CRISPR-Cas9 tools offer unprecedented experimental approaches to dissect the mechanisms of beta cell function in health and disease. They can be used to modulate transcription and manipulate the genome in human beta cell lines [12, 36]. They also enable the generation of novel genetically modified animal models (not only restricted to rodents), allowing comparison and the conservation of beta cell function mechanism across species. CRISPR-Cas9 approaches are also used on hPSCs to generate gene knockouts [42], correct and introduce point mutations [43], engineer fluorescent reporter cell lines and modulate transcription.

## Proof of principle

### Modelling of monogenic diabetes

Monogenic diabetes presents at a young age due to mutations in a single gene, leading to impaired function of the pancreatic beta cells. The exact molecular mechanisms leading to beta cell failure can be addressed in carefully planned cellular models. A particular genetic modification (e.g. knockout, knockin) can be generated in a well-differentiating, healthy hPSC line or a candidate mutation can be corrected in patient-derived hiPSCs. Resulting isogenic cell line pairs have the same genetic background and similar differentiation properties, while being discordant only for the mutation of interest.

Genome editing has been used on hPSCs to knock out genes critical for pancreatic and beta cell development (e.g. *PDX1*, *NEUROG3*, *ARX*, *GLIS3*, *NEUROD1*), thus reproducing with human cells previous findings made in transgenic mouse studies [42, 44, 45]. Furthermore, correction of point mutations in patient-derived hiPSCs has been exploited to interrogate the disease mechanism in rare cases of neonatal diabetes, such as those caused by mutations in *STAT3* and *GATA6* genes [43, 45]. Recently, this strategy was used to show how diabetogenic mutations in the *INS* gene lead to chronic endothelial reticulum stress-associated failure of beta cell growth [46].

### Modelling of polygenic diabetes

More than 400 SNPs associated with type 2 diabetes and related traits have been identified thus far. The functional elucidation of these loci has, however, been largely elusive [1]. For the last 100 years diabetes has been diagnosed by measuring one metabolite, glucose. This has of course identified individuals with elevated glucose levels but provided little information on underlying pathogenic causes. By including six variables (age at diagnosis, BMI, HbA_1c_, GAD autoantibodies, C-peptide and glucose [for estimation of insulin secretion, HOMA-B and insulin-sensitivity, HOMA-IS]) in a clustering analysis of individuals with newly diagnosed diabetes, we could break down classical type 2 diabetes into five distinct subgroups, with better prediction of disease progression and outcome [2]. These clusters also seem to differ in genetic background.

Therefore, gene silencing and functional characterisation or genome editing of type 2 diabetes risk alleles or deleting regulatory regions surrounding these variants in human beta cell models, followed by RNA sequencing (RNAseq) and/or implementation of spatial chromatin organisation methods (4C/Hi-C/Capture-C), could facilitate a better understanding of the functional effects of these variants. In the human beta cell line EndoC-βH1, the silencing of candidate genes selected from 75 type 2 diabetes-associated loci revealed 45 genes involved in beta cell function including *ARL15*, *ZMIZ1*, and *THADA* [47]. Beta cell-specific long non-coding RNA (lncRNA) transcript knockdown and coexpression analysis demonstrated the role of lncRNAs that collaborate with transcription factors to regulate beta cell-specific transcriptional networks. Further, *PLUTO* (also known as *PLUT*), the antisense transcript of the *PDX1* gene, modulates the chromatin structure and transcription of *PDX1*. Both *PDX1* and *PLUTO* are downregulated in islets from hyperglycaemic donors [36]. One of the strongest association signals for type 2 diabetes is the rs7903146 T allele SNP in the *TCF7L2* gene, and researchers have tried hard to understand the molecular mechanism behind this association. Some previous studies have reported increased chromatin accessibility and episomal enhancer activity for the T allele SNP and higher *TCF7L2* expression was found in carriers of the TT genotype with type 2 diabetes [48, 49]. Recently, it was shown that CRISPR-mediated deletion of the region harbouring the type 2 diabetes risk SNP rs7903146 leads to a decrease in *TCF7L2* mRNA levels, while targeting it with a CRISPR transcriptional activator had the opposite effect. These findings further indicate that this region constitutes an enhancer regulating *TCF7L2* expression in human islet cells [12].

The interrogation of type 2 diabetes risk variants could be performed in an unbiased manner by combining CRISPR-Cas9-based genome-wide genome and epigenome editing with single-cell omics to assess the transcriptional and functional outcome of the variants [41]. Genes associated with type 2 diabetes risk have been knocked out in hPSCs to elucidate their putative role and mechanism predisposing to the disease (e.g. *CDKAL1*, *KCNQ1*) [50]. Further improvements on the functionality of stem cell-derived beta-like cells will provide better chances to unravel the functional impact of type 2 diabetes risk variants on beta cell development and physiology. First examples of genome-edited hPSC models to elucidate the role of specific type 2 diabetes-associated SNPs are starting to appear, as exemplified by a report where CRISPR-Cas9-edited hiPSCs and EndoC-βH1 cells were used to investigate the mechanisms of a protective zinc transporter 8 (Znt8, *SLC30A8*) variant [51].

### Outlook

It is easy to predict that the recent technological developments in human cellular models combined with targeted genome modification will lead to a boom in functional genomic studies of diabetes during the coming years. The task is formidable because of the many disease-associated loci in non-coding DNA regions. Ingenious use of CRISPR-Cas9 and similar techniques will undoubtedly speed up the understanding of interplay between type 1 diabetes and type 2 diabetes risk-associated genetic variants and their functional role in predisposing to the disease. These approaches will also be used in drug screens, enhancing the development of targeted means for personalised treatment.

## Electronic supplementary material

ESM 1 (PPTX 209 kb)

## Abbreviations

AAV: Adenovirus and adeno-associated viruses
Cas9: CRISPR-associated protein 9
CRISPR: Clustered Regularly Interspaced Short Palindromic Repeats
gRNA: Guide RNA
GWAS: Genome-wide association studies
HDR: Homology-directed repair
hESC: Human embryonic stem cell
hiPSC: Human induced pluripotent stem cell
hPSC: Human pluripotent stem cell
lncRNA: Long non-coding RNA
