# Supplementary material for: Genome editing of human pancreatic beta cell models: problems, possibilities and outlook
Source: Diabetologia. 2019 Jun 3;62(8):1329–36. doi: 10.1007/s00125-019-4908-z (PMC6647170; doi:10.1007/s00125-019-4908-z)
Supplement: Supplementary file 1 — (PPTX 209 kb) [file 125_2019_4908_MOESM1_ESM.pptx]

## Slide 1
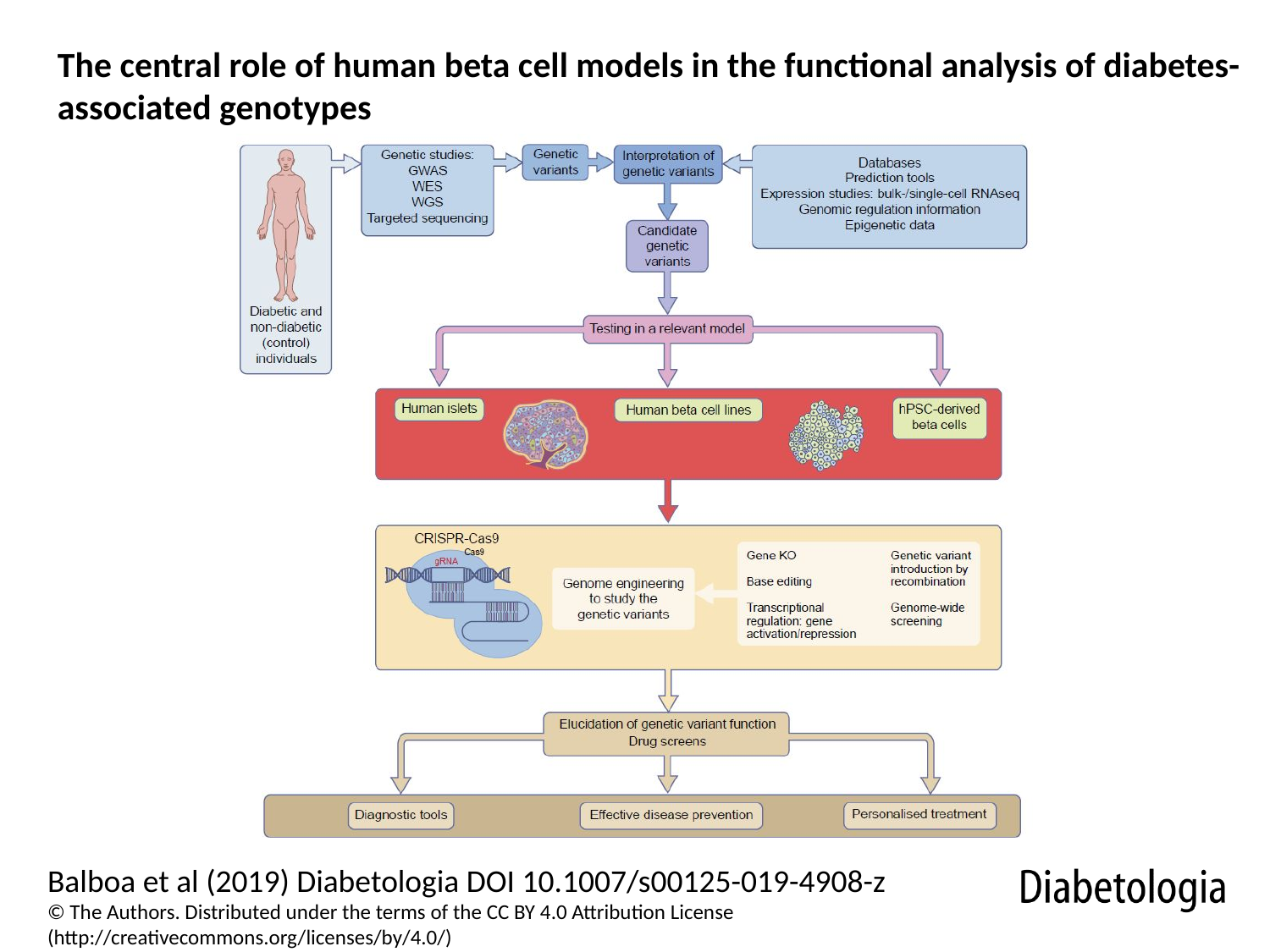

The central role of human beta cell models in the functional analysis of diabetes-associated genotypes
Balboa et al (2019) Diabetologia DOI 10.1007/s00125-019-4908-z
© The Authors. Distributed under the terms of the CC BY 4.0 Attribution License (http://creativecommons.org/licenses/by/4.0/)
